# Progressive Course of Chronic Tick-Borne Encephalitis Manifesting as Amyotrophic Lateral Sclerosis-like Syndrome 35 Years after the Acute Disease

**DOI:** 10.3390/brainsci12081020

**Published:** 2022-07-31

**Authors:** Viktor P. Volok, Larissa V. Gmyl, Ilmira K. Belyaletdinova, Galina G. Karganova, Evgenii P. Dekonenko

**Affiliations:** 1 Chumakov Federal Scientific Center for Research and Development of Immune-and-Biological Products of Russian Academy of Sciences, 108819 Moscow, Russia; volok_vp@chumakovs.su (V.P.V.); gmyl_lv@chumakovs.su (L.V.G.); belyaletdinova_i@mail.ru (I.K.B.); 2 Department of Biology, Lomonosov Moscow State University, 119991 Moscow, Russia; 3 Department of Medical Elementology, Peoples’ Friendship University of Russia (RUDN University), 6 Miklukho-Maklaya St, 117198 Moscow, Russia; 4 Institute of Translational Medicine and Biotechnology, Sechenov Moscow State Medical University, 119991 Moscow, Russia

**Keywords:** tick-borne encephalitis, TBEV, chronic infection, viral infection, amyotrophic lateral sclerosis, ALS-like syndrome

## Abstract

The chronic form of tick-borne encephalitis (TBE) is understudied and seems to be linked exclusively to Siberian and Far Eastern TBE virus (TBEV) subtypes. There are limited clinical descriptions demonstrating that chronic TBE can resemble an amyotrophic lateral sclerosis (ALS)-like disease. Here, we present a description of a clinical case of progressive chronic TBEV infection with a relapse 35 years after the initial acute infection following a tick bite. The disease manifested as an ALS-like syndrome with bulbar signs, progressive muscle weakness and atrophy, decreased reflexes, and eventual respiratory failure and death. There is no clear differentiation between chronic TBE and postencephalitic syndrome described in European sources. The reactivation of TBEV infection was supported by the presence of anti-TBEV antibodies in serum and antibodies to E protein and to the nonstructural protein NS1 in the CSF. These findings support the diagnosis of a relapse of chronic TBE in this patient.

## 1. Introduction

Tick-borne encephalitis (TBE) is a severe infectious disease affecting the central nervous system (CNS) and causing significant morbidity and mortality in the Russian Federation and several countries in Europe and Asia. In the vast majority of cases, TBE is characterized by an acute course, but it can transition into a progressive (chronic) form, which is presumably associated with Siberian and Far Eastern TBE virus subtypes [1]. After acute TBE, a significant proportion of patients experiences long-term neurological and neuropsychological sequelae, referred to as postencephalitic syndrome [2]. There are no generally accepted diagnostic criteria distinguishing postencephalitic syndrome from chronic TBE.

According to Poponnikova et al., in Western Siberia from 1981 to 2003, chronic TBE accounted for 1–3% of the total number of cases [3]. For the earlier periods, Pogodina et al. indicate a similar average frequency, varying between regions [4]. There is some variance in the data on the occurrence of chronic TBE depending on the source, which may be due to the lack of generally accepted diagnostic criteria and significant difficulties in collecting epidemiological data. Many authors note the difficulties in diagnosing the chronic form of TBE due to its similarity with syringomyelia, muscular amyotrophy, ALS, and other conditions [5,6,7].

Depending on the course of the disease, chronic TBE may present as various combinations of periods of progression and stabilization, including primary progressive, relapsing, and secondary progressive forms. Based on clinical symptoms, chronic TBE can be represented by a hyperkinetic syndrome with or without Kozhevnikov’s epilepsy (first described in Russia in 1894, [8]) or an amyotrophic/poliomyelitic form, including ALS-like syndromes [9]. According to Volkova et al., ALS-like disease constituted 3% of a studied cohort with chronic TBE [10].

Amyotrophic lateral sclerosis (ALS) is an incurable progressive degenerative disease of the CNS that leads to paralysis and muscle atrophy. The etiology of ALS is unknown [11]. There is a group of conditions that are clinically similar to ALS but differ in the progression and response to therapy; they are classified as ALS-like (or ALS-mymic) syndromes [12]. ALS-like syndromes are distinguished by the involvement of an earlier age group, subacute course, and stabilization or improvement of the condition during treatment. Several cases of ALS-like syndrome have been associated with viral infections, including human immunodeficiency virus (HIV), human T-lymphotropic virus (HTLV)-1, and chikungunya virus infections [13,14,15,16].

Here, we describe a patient with a secondary progressive form of chronic TBE manifesting as an ALS-like syndrome decades after the initial infection.

## 2. Case Description

Patient L., 56 years old, was admitted to the clinic in 2007 with complaints of pain in the neck, left shoulder, and back, as well as dysarthria and excessive salivation.

The patient had a history of acute TBE at the age of 21, in 1972, when he was in Siberia (Irkutsk region), where he was bitten by an infected tick on his neck. The disease began with high fever, but consciousness was not disturbed. He was hospitalized in the city of Angarsk. CSF analysis showed inflammatory changes, and anti-TBEV antibodies were found in the serum. Later, he developed flaccid paresis of the neck and shoulder girdle with moderate limitation of movements in the cervico-shoulder region. The patient was discharged with residual mild paresis of the neck and shoulder muscles. A year later, in March 1973, the patient was hospitalized in Infectious Clinical Hospital No. 1 (ICH No. 1) in Moscow with complaints of progressive loss of strength in the proximal arms, neck weakness, a decrease in the pharyngeal reflex, and limited mobility of the soft palate. He received symptomatic treatment and did not seek help again after discharge from the hospital. Afterwards he was not receiving any treatment and reported a satisfactory return to all his normal activities. He graduated with a university degree and worked as an engineer. He led an active lifestyle, riding a bike up to 80–100 km a day. He was married and raised a son.

In the summer of 2006, at the age of 55, the patient’s condition began to worsen. The patient again experienced pain in the neck and shoulder girdle, hoarseness of the voice, and speech difficulties. According to his account, the symptoms appeared after a longer-than-usual 120 km bicycle ride. Over the course of several months, his condition progressively worsened; he completely lost his ability to speak and developed profuse salivation and difficulty swallowing. He also experienced periodic episodes of respiratory failure.

**Figure 1:**
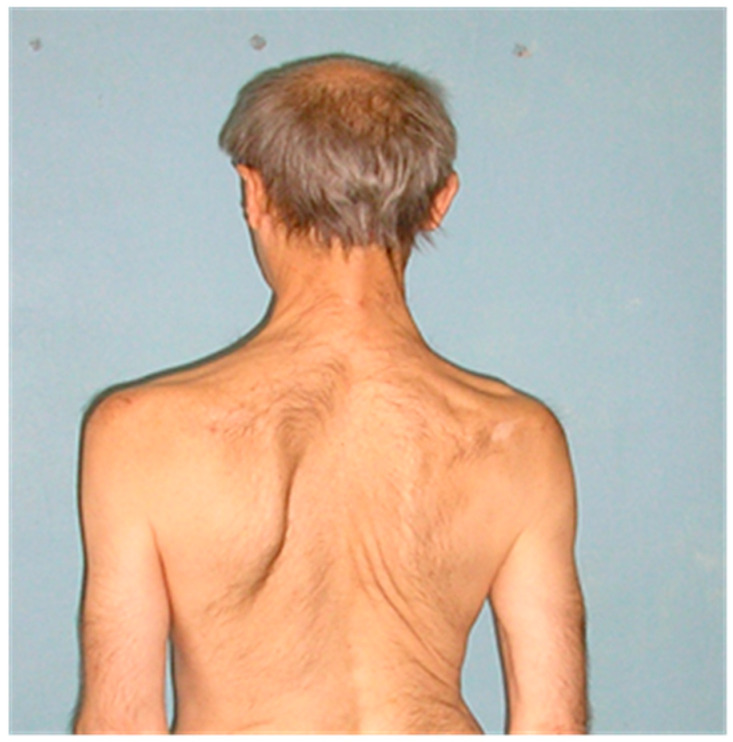
Hypotrophy of the shoulder girdle and neck muscles is clearly visible on the right side. The left scapula is winged.

In the autumn of 2007, he was under observation at the ICH No. 1. Upon neurological examination, his face was hypomimic and symmetrical; eye movements were complete; the tongue was in the oral cavity, flattened, and atrophic, and the patient could not move it beyond the edge of the teeth. Speech production was absent. The pharyngeal reflex was low, and the soft palate had limited mobility. Hypersalivation, swallowing difficulties, and weakness of the masticatory muscles were observed. In his shoulder joints, there was a limitation of mobility, mainly on the left side. Hypotrophy of the shoulder girdle was expressed, more on the right side. A winged scapula was observed on the left side (Figure 1).

The patient’s neck muscles were atrophic, especially mm. sternocleidomastoideus on both sides. His head was hanging forward. Pronounced fasciculations and fibrillations were observed at rest in the proximal muscles of the upper extremities. The strength of the hands in the distal sections was preserved. Tendon reflexes of the hands were not increased. The patient was able to walk independently, and his gait was not impaired. Range of motion and strength in the legs were normal. Sensitivity disorders were not identified.

An EEG showed the focus of epileptiform activity in the right frontal central-parietal region. Clinical and biochemical blood tests were in the normal range. Serum protein electrophoresis was normal. According to the results of CSF analysis, no inflammatory changes were found (protein 0.264 g/L, cytosis 1 cell/μL, glucose 2.4 mmol/L).

**Figure 2:**
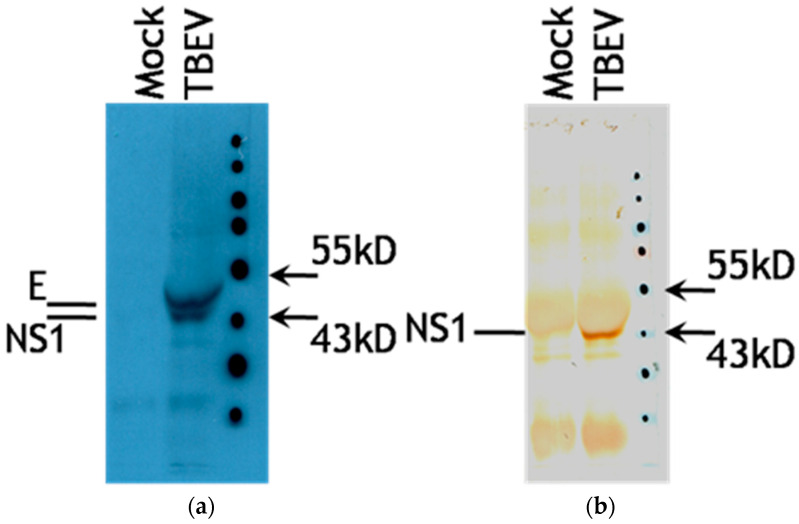
(**a**) TBEV-infected PEK cell culture lysate (TBEV) and lysate of intact cells (Mock) labeled with ^14^C-chlorella hydrolysate were immunoprecipitated with the patient’s CSF on protein A-Sepharose, examined by SDS-PAGE, and developed on X-ray film. E and NS1 bands show the presence of antibodies to these proteins in the CSF. (**b**) Western blot of the obtained immunoprecipitate stained with a polyclonal rabbit serum to NS1 protein of TBEV.

Anti-TBEV antibodies were detected in the blood serum with a titer of 1:800 measured by ELISA by the method of limiting dilutions. ELISA was performed using TBEV IgG/IgM kits (Vector-Best, Russia). Antibodies in the CSF were analyzed by immunoprecipitation of viral proteins from radiolabeled lysates of TBEV-infected porcine embryo kidney (PEK) cells [17]. The presence of antibodies to proteins E and NS1 in the patient’s CSF was confirmed. The specificity of antibodies to NS1 protein was supported by Western blotting of the immunoprecipitate with rabbit polyclonal serum to protein NS1 (Figure 2).

The patient received symptomatic treatment, which was recommended for management of neuromuscular conditions by local medical regulators at the time (neostigmine, nootropics, ATP injections, and nandrolone), as well as antivirals (ribavirin, interferon alpha), without effect. The patient died of progressive respiratory failure.

## 3. Discussion

The presented case history of a patient with actively progressing bulbar dysfunctions has some similarities to the bulbar form of amyotrophic lateral sclerosis (ALS). The patient had suffered an acute form of TBE 35 years before admission to our clinic. Despite having had residual mild atrophies of the shoulder and neck muscles, in subsequent years, he felt well, did not seek medical help for this problem, and led an active lifestyle.

Several decades after the disease, presumably after a significant overstrain, the patient developed severe impairment of cerebral functions with bulbar symptoms resembling ALS-like syndrome. It is known that chronic viral infections can be reactivated under the influence of intense physical or psychological stress, hypothermia, or concomitant infectious disease [7,18,19]. The recurrence of chronic TBE due to physical stress was also described [2]; however, it is not possible to unequivocally establish the cause of relapse in the case of this patient.

Many authors note the difficulties in diagnosing the chronic form of TBE due to its similarity with conditions such as syringomyelia, muscular amyotrophy, ALS, and others [5,6,7]. According to the disease course, there are two types of chronic TBE: a recurrent type, with alternating periods of deterioration and stabilization; and a progressive course, with a steady decline of the condition [5,7]. The latter is subdivided into a hyperkinetic form manifesting as a so-called Kozhevnikov epilepsy and an amyotrophic form, which is characterized by ALS-like syndrome [9]. The transition to a chronic course is more often observed when the disease starts as a focal TBE with poliomyelitis syndrome of cervicobrachial localization (as in our patient) [20]. According to the literature, the new symptoms developed predominantly after several months (in most cases, up to 1 year) or years (3–5 years) after the acute disease [5]; however, cases have been reported with the relapse 5 to 19 years after the acute period [7,21]. In summary, the development of an ALS-like syndrome in chronic TBE after a focal form of TBE with poliomyelitis syndrome has been described by several authors, which is consistent with our observations [6,22,23]. It should be noted that even for classical ALS, there are significant difficulties in conceptualization and diagnosis [24], whereas for ALS-like syndromes, no universally accepted diagnostic guidelines exist. Considering these limitations, this work contributes to our collective knowledge about virus-induced ALS-mimicking conditions. Unfortunately, we were unable to use nerve conduction studies or MRI for this patient. These methods would allow us to explain the observed neurological findings and to better understand the pathophysiology of the amyotrophic form of chronic TBE.

Another issue worth discussing is the differential diagnosis of chronic TBE and postencephalitic syndrome. Chronic TBE as a diagnosis has been described only in sources from the former Soviet Union, as it is probably associated the Siberian and Far Eastern TBEV subtypes, which are not common in Europe [21]. Furthermore, European studies of long-term TBE sequelae were clinical and epidemiological in nature and did not aim to establish or refute the presence of persistent TBEV infection in the CNS of the examined patients. For example, in a study by Mickienė et al., 46.2% of patients with TBE of varying severity showed incomplete recovery one year after the disease, and 2 of 133 patients were diagnosed with chronic TBE, although the pathogenesis of these cases was not investigated, and patients recovered after administration of glucocorticosteroids and plasmapheresis [25].

It can be assumed that postencephalitic syndrome develops after the end of the infection and complete elimination of TBEV from the organism and that the residual lesions of the nervous system are mediated by immunopathological processes, whereas chronic TBE, by definition, is characterized by long-term persistence of the virus in the human body, particularly in the CNS. Although immunologically mediated damage cannot be excluded from the pathology of chronic TBE, it is most likely linked to the direct effects of viral reproduction.

It cannot be ruled out that some cases of postencephalitic syndrome observed in patients by European specialists were actually underdiagnosed cases of chronic TBE. On the other hand, some chronic TBE cases diagnosed by Russian doctors could actually be manifestations of postencephalitic syndrome without actual virus persistence, as some of these cases did not have a solid verification of the ongoing infection. In addition to a certain tendency of Russian clinicians to explain the presence of residual symptoms by chronic infections, viral replication during persistence can be restricted in magnitude and/or locality and thus may not be noticeable in serological tests or in the CSF. The detection of viral RNA in the CSF of TBE patients is very rare even with the most sensitive assays, and the absence of TBEV RNA in the CSF does not exclude the presence of active infection [26].

Considering the epidemiological, clinical, and laboratory data obtained during the examination, we diagnosed the patient with secondary progressive chronic TBE manifesting as an ALS-like syndrome with bulbar involvement. Serological analysis demonstrated the presence of anti-TBEV antibodies in the blood, as well as the presence of antibodies to viral glycoprotein E and, crucially, to non-structural protein NS1 in the CSF, which is considered to indicate an active TBEV infection in the CNS [1]. The validity of our diagnosis is indicated, among other things, by the fact that the relapse was topically very similar to the acute TBE the patient had endured previously; in particular, the cervical and shoulder regions were affected again. However, during the relapse, the neurological damage was more severe, widespread, and eventually fatal.

We cannot fully exclude the possibility of reinfection with TBEV in this case. To the best of our knowledge, no cases of reinfection with TBEV in people surviving clinically manifest disease have been documented. The immune response after the clinically obvious TBE the patient had endured should have been very strong, but in the absence of data, we cannot be sure that titers of anti-TBE IgG would be high enough to protect him from reinfection with TBEV after 30+ years.

Even if we accept the hypothetical possibility of such reinfection in this case, we believe it is highly unlikely because of a combination of several factors. First, the patient’s own report that he had not been bitten by ticks, had not been to forests recently, and had not travelled lately to other regions (in 2006, there were no detected TBEV cases or reported circulation of the virus in Moscow and Moscow region). Second, the clinical course of acute TBE usually has a well-defined pattern, which was absent in this case, and the relapse was similar to the one that happened a year after the acute infection. Third, the fact that the same regions of the body were affected during the relapse (neck, shoulders, throat, etc.) points to the reactivation of the previous infection.

Unfortunately, all therapeutic measures directed towards minimizing the neuromuscular symptoms were ineffective, and the disease quickly progressed to respiratory failure and death.

The defining feature of this clinical case is an extremely long period between the acute disease and the reactivation and relapse of the persistent TBEV infection. It is known that the onset of progressive chronic TBE is most often observed during the first year after the acute disease, although in some cases, this period lasted as long as 19 years [5,7,21]. Nevertheless, to the best of our knowledge, this is the first laboratory-confirmed case of progressive TBE with an asymptomatic period lasting as long as 35 years.

Further research is necessary in order to develop effective treatments for the neurological complications of TBE mediated either by viral persistence or by immunopathology. Because in some patients with HIV-induced ALS-like syndrome, antiretroviral therapy has resulted in resolution of symptoms [27], it is possible that a specific antiviral therapy could be effective against ALS-like disease caused by TBEV.

## Data Availability

Not applicable.
